# Combined Effect of Retinoic Acid and Basic Fibroblast Growth Factor on Maturation of Mouse Oocyte and Subsequent Fertilization and Development 

**DOI:** 10.22074/ijfs.2018.5293

**Published:** 2018-01-05

**Authors:** Morteza Abouzaripour, Fardin Fathi, Erfan Daneshi, Keywan Mortezaee, Mohammad Jafar Rezaie, Mahdad Abdi

**Affiliations:** 1 Cellular and Molecular Research Center, Kurdistan University of Medical Sciences, Sanandaj, Iran; 2 Department of Anatomy, School of Medicine, Kurdistan University of Medical Sciences, Sanandaj, Iran

**Keywords:** Basic Fibroblast Growth Factor, *In Vitro* Maturation, Oocyte, Retinoic Acid

## Abstract

**Background:**

Many autocrine and paracrine elements that are produced within follicular niche have been the focus of much *in vitro* maturation (IVM) research. The present study was carried out to compare retinoic acid (RA) and basic fibroblast growth factor (bFGF) efficacy on IVM of mouse oocytes, and their further dual consumption to reach an optimal protocol.

**Materials and Methods:**

In this experimental study, germinal vesicle (GV) oocytes obtained from two-months-old NMRI mice were randomly divided into control, sham and three experimental groups. The basic culture medium was α-MEM supplemented with 10% fetal bovine serum (FBS), 50 mg/l streptomycin, 60 mg/l penicillin and 10 ng/ ml epidermal growth factors. Each of the experimental groups received one of the following treatments: RA (2 µM), bFGF (20 ng/ml) or combination of RA and bFGF with the indicated concentrations. After 24 hours, capacitated spermatozoa were added to *in vitro* matured oocytes. Five hours later, the oocytes were cultured in fresh droplets of M2 medium for 24 hours and assessed for cleavage to the two-cells stage.

**Results:**

As compared with the control group, the rate of maturation was significantly increased in the RA (P<0.001) and bFGF+RA (P<0.02) groups with 58 ± 10 and 57 ± 3.46, respectively. The rate of maturation was significant in the RA (P<0.02) and bFGF+RA (P<0.03) groups, in comparison with the bFGF group. The bFGF+RA group had higher rate (83 ± 1.52) of two-cells development, than control (33 ± 1, P<0.001).

**Conclusion:**

Our findings showed beneficial effects of 2 µM RA and 20 ng/ml bFGF combination on mouse oocyte IVM.

## Introduction

In spite of great scientific breakthrough for *in vitro* maturation (IVM), the number of mature oocytes obtained from these methods and their fertilization rates is still too low. So, researchers are trying to achieve a superior approach for in vivo recapitulation of follicular environment. Thus far, many elements have been surveyed to assess oocyte maturation within follicular niche. Supplementation of maturation medium with various complements is a promising method. The active form of vitamin A, retinoic acid (RA), is an example of these complements involved in very initial events of mammalian reproduction, including follicular growth, oocyte maturation, embryonic growth and its development (1).

Positive effects of RA on IVM of cumulus oocyte complexes have previously been described (2-5). Fibroblast growth factors (FGFs) produced by theca and granulosa cells are involved in diverse biological processes during folliculogenesis, but the role of these factors during the ultimate period of oocyte maturation remained yet unknown (6). The present study was accomplished to survey the combined role of RA and bFGF in IVM of mouse oocytes to reach an optimal protocol. We propose that providing dual supplementation of maturation medium with RA and bFGF during IVM may probably be beneficial for oocyte maturation and the subsequent embryo development.

## Materials and Methods

In this experimental study, the animals were kept under controlled conditions (12 hour light: 12 hour dark), fed with water ad libitum. All procedures were performed in accordance with the approval of the Institutional Animal Care and Use Committee at the Kurdistan University of Medical Sciences (MUK, Iran). All reagents were purchased from Sigma-Aldrich Co, USA.

**Table 1:** Outcome of oocytes IVM in different groups


Group	GV numbersn	Arrested GV Mean ± SD	Degenerated GV Mean ± SD	GVBD Mean ± SD	MII Mean ± SD

Control	110	39.33 ± 2.08	10 ± 4	30.33 ± 1.52	31.66 ± 1.52
sham (ethanol)	120	41.33 ± 1.15	13.66 ± 2.30	34 ± 1.73	32.66 ± 0.57
bFGF	115	18.33 ± 1.52	12.33 ± 1.15	44.66 ± 3.21	40.66 ± 2.30
RA	125	16.33 ± 0.57	4.33 ± 0.57	47 ± 2	58 ± 1^$,@^
bFGF+RA	120	17.33 ± 2.30	6.33 ± 0.57	38.66 ± 1.15	57 ± 3.46^*, #^


*; P<0.03 vs. bFGF, #; P<0.02 vs. control, $; P<0.02 vs. bFGF, @; P<0.001 vs. control and sham, IVM; In vitro maturation, GV; Germinal vesicle, GVBD; GV break down, MII; Miosis phase II, bFGF; Basic fibroblast growth factor, and RA; Retinoic acid.

### Collection of immature mouse oocytes

Animals were superovulated by an intraperitoneal injection of 10 IU pregnant mare’s serum gonadotropin (PMSG). Mice were sacrificed 44 hours later by cervical dislocation and their ovaries were placed in a-MEM culture medium supplemented with 10% fetal bovine serum (FBS). Immature oocytes in the germinal vesicle (GV) stage were mechanically dissected using 26-G needles attached to a 1 ml syringe under a stereo microscope (Olympus, Japan). The collected GV-stage oocytes obtained from 2-months-old NMRI mice were randomly divided into control, sham and three experimental groups (7).

### *In vitro* maturation

The collected GV-stage oocytes of each group were placed in 25 µl drops of maturation medium consisting of a-MEM supplemented with 10% FBS, 50 mg/l streptomycin, 60 mg/l penicillin and 10 ng/ml epidermal growth factors (EGF), and then they were incubated in a humidified atmosphere of 5% CO_2_ at 37°C for 24 hours. 

In the first experimental group, maturation medium was incubated with 2 µM RA dissolved in pure ethanol (8), and in the second experimental group, it was incubated with 20 ng/ml bFGF (9). In the third experimental group, combined RA and bFGF with the same concentrations was added to the maturation medium. In the sham group, 0.2% (v/v) ethanol was added to the maturation medium. After 24 hours, oocytes were observed under inverted microscope. Nuclear maturation of GV stage was determined by evaluation of morphological changes in the nucleus or appearance of the first polar body (MΙΙ). Matured oocytes were collected and used for *in vitro* fertilization (IVF).

### *In vitro* culture and *in vitro* fertilization

Sperms of 12-weeks-old male NMRI mice were collected from the tail of epididymis. Sperm suspension (1×10^6^ motile spermatozoa/ml) was capacitated for 1 hour in 500 µl human tubular fluid (HTF) culture medium. *In vitro* matured oocytes from each group were added to 100 µl droplets of HTF to which 0.1 ml of capacitated spermatozoa was added. After 5 hours of incubation, the oocytes were washed with three droplets of HTF medium and checked for appearance of the second polar body and formation of male and female pronuclei indicating fertilization. Then, oocytes were cultured in fresh droplets of M2 medium (25 µl) covered by mineral oil and assessed for cleavage to the two-cells stage after 24 hours (1).

### Statistical analysis

Data were analyzed using One-way ANOVA with a post- hoc Tukey and presented as mean ± SD. The differences in the values of maturation, fertilization and developmental rates were considered significant at P<0.05. All computations were carried out using SPSS 16 for Windows.

## Results

### *In vitro* maturation of mouse oocytes

**Fig.1:**
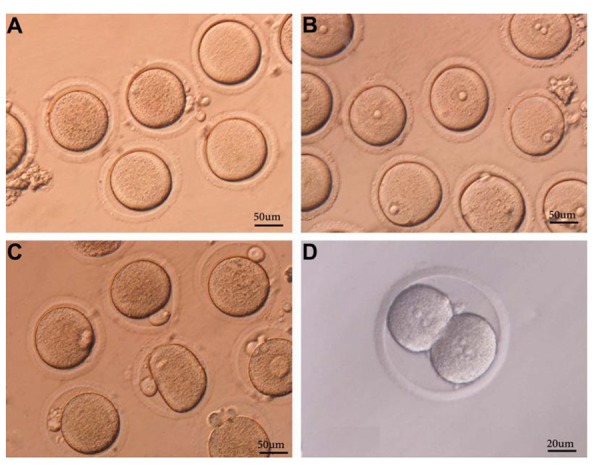
Oocytes in various stages of development. A. Germinal vesicle break down (GVBD), B. GV, C. Mature oocytes with polar bodies, and D. Two-cells stage.

Development of oocytes from GV break down (GVBD) to two-cells stage has been shown in in the Figure 1. The maturation rate of cultured GV-stage oocytes was low in both control and sham groups with 31.66 ± 1.52 and 32.66 ± 0.57, respectively. As compared with the control group, the rate of maturation was significantly increased in the RA (P<0.001) and bFGF+RA (P<0.002) groups with 58 ± 1 and 57 ± 3.46, respectively. The rate of maturation was significant in the RA (P<0.02) and bFGF+RA (P<0.03) groups compared to the bFGF group (Table 1).

### *In vitro* fertilization and development of mouse oocytes

Data from Table 2 showed that the bFGF+RA group had a higher rate 83 ± 1.52 (47.7%) of two-cells development, compared to the control 33 ± 1 (34%) (P<0.001). The number was significant in the bFGF+RA group in comparison with the bFGF (P<0.001, Table 2).

**Table 2:** The number and percentage of oocytes attaining the two-cells stage after 24 hours of culture


Group	Number of MIIn	Number of two-cells stage Mean ± SD (%)

Control	95	33 ± 1 (34)
sham (ethanol)	65	20 ± 0.57 (30)
bFGF	122	51 ± 1 (41)^#^
RA	174	58 ± 0.57 (50)^*^
bFGF+RA	116	83 ± 1.52 (47.7)^*^


*; P<0.001 vs. bFGF, sham and control, #; P<0.001 vs. all groups, MII; Miosis phase II, bFGF; Basic fibroblast growth factor, and RA; Retinoic acid.

## Discussion

In the present survey, we compared the effect of RA and bFGF on maturation of mouse oocytes and their further development into two-cells stage. We found that separate usage of either RA or bFGF in basic culture medium could improve outcomes of IVM. Achieving an efficient culture system for IVM is an important criterion in reproductive research. The advantageous roles of retinol metabolites in *in vitro* cytoplasmic maturation and embryonic development have formerly been demonstrated (10, 11). Previous studies reported that RA may stimulate follicle-stimulating hormone (FSH) for induction of luteinizing hormone (LH) receptors RA regulates progesterone generation and reduces cAMP levels (12). It could also protect oocyte against oxidative stress induced by apoptosis (13, 14) through reduction of free oxygen radicals and interaction with other antioxidant compounds (15).

bFGF has been known as an oocyte competency factor due to its formation from theca, granulosa and cumulus cells throughout folliculogenesis (16). Researchers asserted that bFGF is localized in the primordial and early developing follicles, and that this growth factor stimulates primordial follicle development and further cell growth (17). Addition of bFGF to the medium has also been shown to be beneficial in improvement of oocyte development (18, 19). We found an increase in the number of oocytes attaining two-cells stage after addition of bFGF to the medium for 24 hours. This number was considerably lower compared to the RA group. When combination of RA and bFGF was used, there were no significant changes compared to the RA group. Therefore we propose that both RA and bFGF could improve IVM quality, and the role of RA was more noticeable than that of bFGF to develop into two-cells stage.

## Conclusion

Our findings showed beneficial effects of 2 µM RA and 20 ng/ml bFGF on mouse oocyte IVM.

## References

[1] Pu Y, Wang Z, Bian Y, Zhang F, Yang P, Li Y (2014). All-trans retinoic acid improves goat oocyte nuclear maturation and reduces apoptotic cumulus cells during in vitro maturation. Anim Sci J.

[2] Hidalgo CO, Díez C, Duque P, Facal N, Gómez E (2003). Pregnancies and improved early embryonic development with bovine oocytes matured in vitro with 9-cis-retinoic acid. Reproduction.

[3] Almiñana C, Gil MA, Cuello C, Caballero I, Roca J, Vazquez JM (2008). In vitro maturation of porcine oocytes with retinoids improves embryonic development. Reprod Fertil Dev.

[4] Duque P, Díez C, Royo L, Lorenzo PL, Carneiro G, Hidalgo CO (2002). Enhancement of developmental capacity of meiotically inhibited bovine oocytes by retinoic acid. Hum Reprod.

[5] Lima PF, Oliveira MA, Santos MH, Reichenbach HD, Weppert M, Paula-Lopes FF (2006). Effect of retinoids and growth factor on in vitro bovine embryos produced under chemically defined conditions. Anim Reprod Sci.

[6] Zhang K, Ealy AD (2012). Supplementing fibroblast growth factor 2 during bovine oocyte in vitro maturation promotes subsequent embryonic development. Open J Anim Sci.

[7] Abedelahi A, Salehnia M, Allameh AA (2008). The effects of different concentrations of sodium selenite on the in vitro maturation of preantral follicles in serum-free and serum supplemented media. J Assist Reprod Genet.

[8] Tahaei LS, Eimani H, Yazdi PE, Ebrahimi B, Fathi R (2011). Effects of retinoic acid on maturation of immature mouse oocytes in the presence and absence of a granulosa cell co-culture system. J Assist Reprod Genet.

[9] Ghasemian F, Bahadori MH, Azarnia M, Soltani B, Nasiri E, Ahmadi M (2010). The beneficial effect of fibroblastic growth factor on in vitro maturation and development ability of immature mouse oocytes. The Open Reproductive Science Journal.

[10] Gómez E, Royo LJ, Duque P, Carneiro G, Hidalgo C, Goyache F (2003). 9-cis-retinoic acid during in vitro maturation improves development of the bovine oocyte and increases midkine but not IGF-I expression in cumulus-granulosa cells. Mol Reprod Dev.

[11] Lonergan P, Gutiérrez-Adán A, Rizos D, Pintado B, de la Fuente J, Boland MP (2003). Relative messenger RNA abundance in bovine oocytes collected in vitro or in vivo before and 20 hr after the preovulatory luteinizing hormone surge. Mol Reprod Dev.

[12] Bagavandoss P, Midgley AR Jr (1988). Biphasic action of retinoids on gonadotropin receptor induction in rat granulosa cells in vitro. Life Sci.

[13] Ahlemeyer B, Bauerbach E, Plath M, Steuber M, Heers C, Tegtmeier F (2001). Retinoic acid reduces apoptosis and oxidative stress by preservation of SOD protein level. Free Radic Biol Med.

[14] Guérin P, El Mouatassim S, Ménézo Y (2001). Oxidative stress and protection against reactive oxygen species in the pre-implantation embryo and its surroundings. Hum Reprod Update.

[15] Moreno-Manzano V, Ishikawa Y, Lucio-Cazana J, Kitamura M (1999). Suppression of apoptosis by all-trans-retinoic acid.Dual intervention in the c-Jun n-terminal kinase-AP-1 pathway. J Biol Chem.

[16] Nilsson E, Parrott JA, Skinner MK (2001). Basic fibroblast growth factor induces primordial follicle development and initiates folliculogenesis. Mol Cell Endocrinol.

[17] Berisha B, Steffl M, Amselgruber W, Schams D (2006). Changes in fibroblast growth factor 2 and its receptors in bovine follicles before and after GnRH application and after ovulation. Reproduction.

[18] Gupta PSP, Ravindranatha BM, Nandi S, Sarma PV (2002). In vitro maturation of buffalo oocytes with epidermal growth factor and fibroblast growth factor. Indian J Anim Sci.

[19] Mondal S, Mor A, Reddy IJ, Nandi S, Parameswaragupta P (2015). Effect of fibroblast growth factor 2 (fgf2) and insulin transferrin selenium (ITS) on in vitro maturation, fertilization and embryo development in sheep. Braz Arch Biol Technol.

